# Supplementary material for: Polypharmacy and the Change of Self-Rated Health in Community-Dwelling Older Adults
Source: Int J Environ Res Public Health. 2023 Feb 25;20(5):4159. doi: 10.3390/ijerph20054159 (PMC10002126; doi:10.3390/ijerph20054159)
Supplement: Supplementary file 1 [file ijerph-20-04159-s001.zip › ijerph-2174166-supplementary.pdf]

Supplemental Material

Polypharmacy and the change of self-rated health in community dwelling older adults

Table S1: Self-rated health of the study population at baseline by polypharmacy status.

Table S2: Baseline characteristics of excluded individuals compared to included participants.

Table S3: Median (IQR) number of medications and comorbidities in individuals with polypharmacy during both study visits stratified by SRH-change category

Table S4: Multinomial regression model showing the association between polypharmacy and SRH-change categories in male participants.

Table S5: Multinomial regression model showing the association between polypharmacy and SRH-change categories in female participants.

Figure S1: Flowchart showing the number of participants included in the current analysis

Figure S2: Distribution of self-rated health (SRH) at baseline (top part) of participants A) without polypharmacy and B) with polypharmacy and their assignment to SRH-change categories (bottom part) over the period of four years.

Table S1. Self-rated health of the study population at baseline by polypharmacy status.

Variable	Category	Polypharmacy	No Polypharmacy	Total
		672 (47.1%)	756 (52.9%)	(N= 1,428)
SRH Level at baseline N (%)	Very Good	16 (2.4%)	44 (5.8%)	60 (4.2%)
	Good	238 (35.4%)	475 (62.8%)	713 (49.9%)
	Moderate	332 (49.4%)	205 (27.1%)	537 (37.6%)
	Poor	77 (11.5%)	29 (3.8%)	106 (7.4%)
	Very Poor	9 (1.3%)	3 (0.4%)	12 (0.8%)

SD: Standard Deviation
SRH: Self-rated Health

Table S2. Baseline characteristics of excluded individuals compared to included participants.

Variable	Category	Exclusion Status		Total
		Included (n = 1,428)	Excluded (n = 641)	(n = 2,069)
Sociodemographic Factors				
Age Mean (SD)		79.1 (6.1)	83.3 (7.1)	80.4 (6.7)
Gender N (%)	Female	771 (54.0%)	317 (49.5%)	1,088 (52.6%)
SRH Level at baseline N (%)	Good	773 (54.1%)	292 (45.6%)	1,065 (51.5%)
	Moderate	537 (37.6%)	245 (38.2%)	782 (37.8%)
	Poor	118 (8.3%)	89 (13.9%)	207 (10.0%)
	Missing	0	15 (2.3%)	15 (0.7%)
Polypharmacy N (%)	Yes	672 (47.1%)	364 (56.8%)	1,036 (50.1%)
	Missing	0	4 (0.6%)	4 (0.2%)
Income (in EUR) N (%)	<1000	409 (28.6%)	165 (25.7%)	574 (27.7%)
	1000 – 1999	754 (52.8%)	317 (49.5%)	1,071 (51.8%)
	≥2000	80 (5.6%)	37 (5.8%)	117 (5.7%)
	Missing	185 (13.0%)	122 (19.0%)	307 (14.8%)
CASMIN N (%)	Low	847 (59.3%)	397 (61.9%)	1,244 (60.1%)
	Intermediate	288 (20.2%)	123 (19.2%)	411 (19.9%)
	High	286 (20.0%)	119 (18.6%)	405 (19.6%)
	Missing	7 (0.5%)	2 (0.3%)	9 (0.4%)
Having a partner N (%)	Yes	860 (60.2%)	351 (54.8%)	1,211 (58.5%)
	Missing	0	2 (0.3%)	2 (0.1%)
Lifestyle Factors				
Frequency of Alcohol Consumption N (%)	Less than once a month	607 (42.5%)	307 (47.9%)	914 (44.2%)
	≤2 times per week	521 (36.5%)	203 (31.7%)	724 (35.0%)
	Regularly	296 (20.7%)	118 (18.4%)	414 (20.0%)
	Missing	4 (0.3%)	13 (2.0%)	17 (0.8%)
Physical Activity N (%)	Less than once a week	312 (21.8%)	215 (33.5%)	527 (25.5%)
	1-5 times per week	681 (47.7%)	280 (43.7%)	961 (46.4%)
	More than 5 times per week	433 (30.3%)	143 (22.3%)	576 (27.8%)
	Missing	2 (0.1%)	3 (0.5%)	5 (0.2%)
BMI (in kg/m^2) N (%)	<25	355 (24.9%)	219 (34.2%)	574 (27.7%)
	25 - <30	688 (48.2%)	260 (40.6%)	948 (45.8%)

	≥30	384 (26.9%)	162 (25.3%)	546 (26.4%)
	Missing	1 (0.1%)	0	1 (0.001%)
Medical Status				
CCI Category N (%)	0	138 (9.7%)	43 (6.7%)	181 (8.7%)
	1-2	410 (28.7%)	129 (20.1%)	539 (26.1%)
	3-4	372 (26.1%)	153 (23.9%)	525 (25.4%)
	≥5	496 (34.7%)	311 (48.5%)	807 (39.0%)
	Missing	12 (0.8%)	5 (0.8%)	17 (0.8%)

SD: Standard Deviation
SRH: Self-rated Health
CASMIN: Comparative Analysis of Social Mobility in Industrial Nations
BMI: Body Mass Index
CCI: Charlson Comorbidity Index

Table S3. Median (IQR) number of medications and comorbidities in individuals with polypharmacy during both study visits stratified by SRH-change category.

	**Stable High**	**Stable Moderate**	**Stable Low**	**Decline**	**Improvement**
Median (IQR) number of medications					
SRH Baseline	6 (5, 7)	7 (5.25, 9)	8 (7, 9)	7 (6, 8)	8 (6, 9)
SRH Follow-up	7 (6, 8)	8 (6, 10)	9 (7, 12)	8 (6, 10)	8 (6, 10)
Median (IQR) number of comorbidities					
SRH Baseline	4 (2, 7)	5 (3, 7)	6 (4, 8)	5 (3, 7)	6 (4, 8)
SRH Follow-up	6 (4, 8.25)	7 (5, 9)	8 (6, 11)	7 (5, 9)	7 (6, 9)

IQR: Interquartile Range
SRH: Self-rated Health

Table S4. Multinomial regression model showing the association between polypharmacy and SRH-change categories in male participants.

	**SRH-Change Categories**				
	**Stable High**	**Stable Moderate**	**Stable Low**	**Decline**	**Improvement**
N (%)					
Polypharmacy					
Yes	78 (23.9)	74 (22.6)	15 (4.6)	95 (29.1)	65 (19.9)
No	164 (49.7)	28 (8.5)	3 (0.9)	97 (29.4)	38 (11.5)
Crude Model OR (95% CI)					
Polypharmacy (Yes)	Reference	5.56 (3.33 – 9.27)	10.5 (2.96 – 37.4)	2.06 (1.39 – 3.04)	3.60 (2.22 – 5.83)
Adjusted Model 1[a] OR (95% CI)					
Polypharmacy (Yes)	Reference	3.76 (2.07 – 6.86)	3.19 (0.79 – 12.9)	1.30 (0.81 – 2.10)	2.58 (1.42 – 4.67)
Adjusted Model 2[b]					

OR (95% CI)					
Polypharmacy (Yes)	Reference	3.73 (2.04 – 6.80)	3.14 (0.78 – 12.70)	1.33 (0.82 – 2.15)	2.56 (1.41 – 4.65)

[a]Adjusted for income, Comparative Analysis of Social Mobility in Industrial Nations (CASMIN), partner status, frequency of alcohol consumption, physical activity, Body Mass Index (BMI), Charlson Comorbidity Index (CCI).
[b]Adjusted model 1 + age

Table S5. Multinomial regression model showing the association between polypharmacy and SRH-change categories in female participants.

	**SRH-Change Categories**				
	**Stable High**	**Stable Moderate**	**Stable Low**	**Decline**	**Improvement**
N (%)					
Polypharmacy					
Yes	52 (15.1)	111 (32.2)	28 (8.1)	110 (31.9)	44 (12.8)
No	185 (43.4)	74 (17.4)	13 (3.1)	106 (24.9)	48 (11.3)
Crude Model OR (95% CI)					
Polypharmacy (Yes)	Reference	5.34 (3.49 – 8.17)	7.66 (3.71 – 15.8)	3.69 (2.46 – 5.55)	3.26 (1.95 – 5.44)
Adjusted Model 1[a] OR (95% CI)					
Polypharmacy (Yes)	Reference	3.43 (2.04 – 5.77)	3.44 (1.46 – 8.11)	2.64 (1.62 – 4.30)	1.51 (0.82 – 2.80)
Adjusted Model 2[b] OR (95% CI)					
Polypharmacy (Yes)	Reference	3.36 (2.00 – 5.67)	3.47 (1.46 – 8.24)	2.48 (1.52 – 4.07)	1.56 (0.84 – 2.90)

[a]Adjusted for income, Comparative Analysis of Social Mobility in Industrial Nations (CASMIN), partner status, frequency of alcohol consumption, physical activity, Body Mass Index (BMI), Charlson Comorbidity Index (CCI).
[b]Adjusted model 1 + age

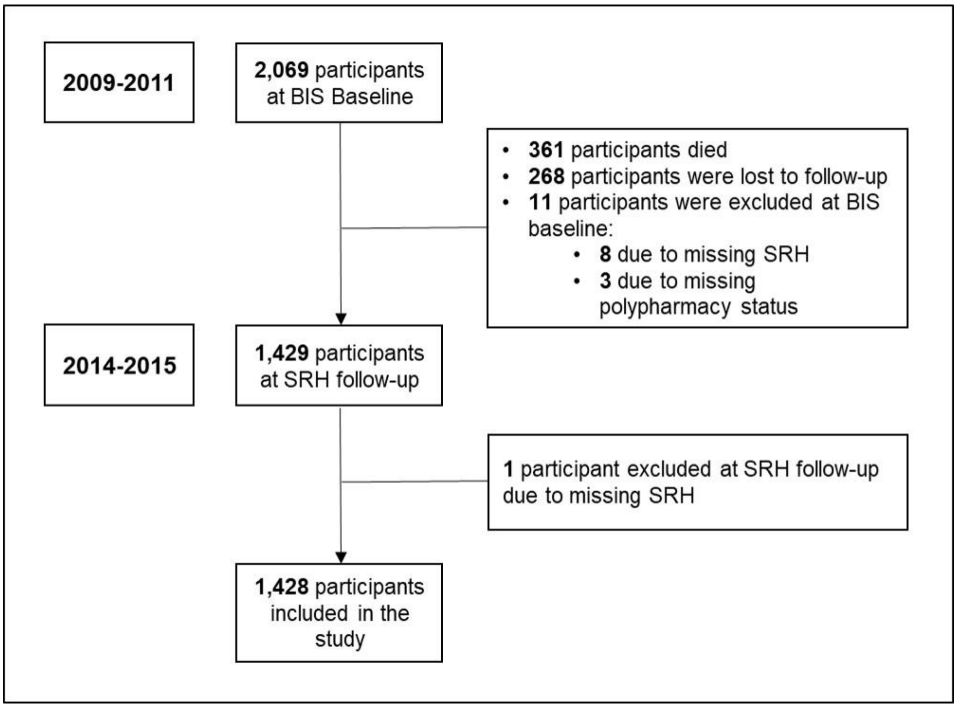

Figure S1: Flowchart showing the number of participants included in the current analysis

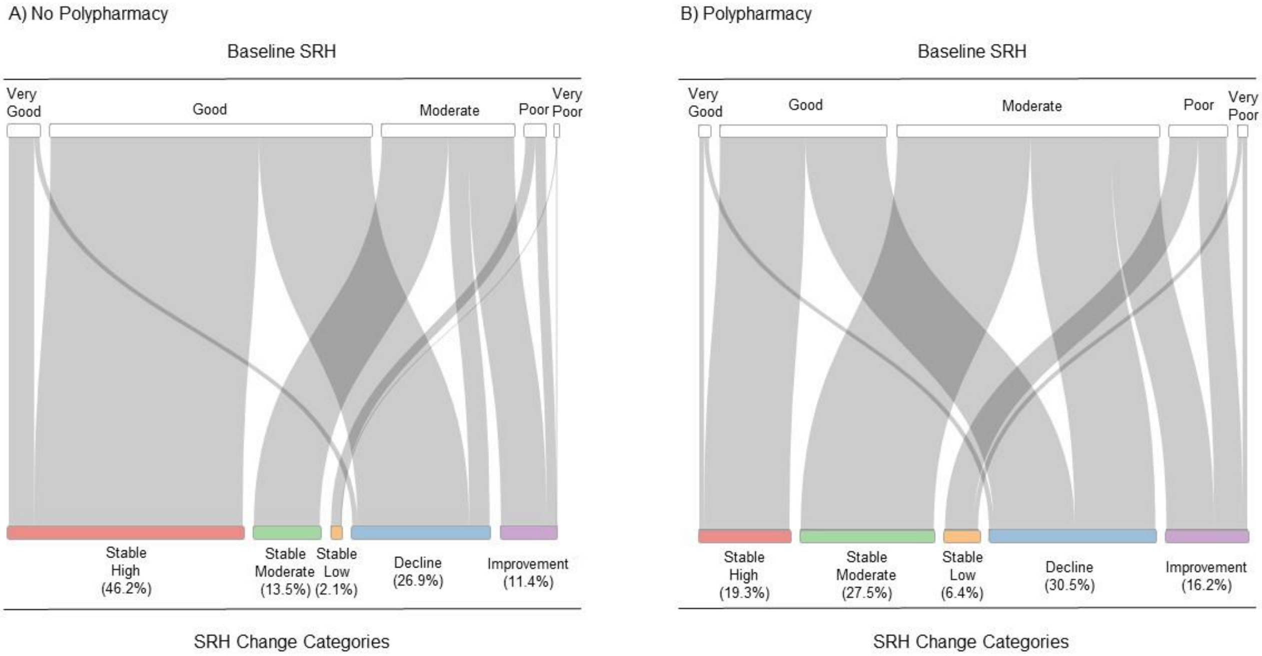

Figure S2: Distribution of self-rated health (SRH) at baseline (top part) of participants A) without polypharmacy and B) with polypharmacy and their assignment to SRH-change categories (bottom part) over the period of four years.
